# The significance of Notch ligand expression in the peripheral blood of children with hand, foot and mouth disease (HFMD)

**DOI:** 10.1186/1471-2334-14-337

**Published:** 2014-06-17

**Authors:** Zhen Jiang Bai, Yi Ping Li, Jie Huang, Yong Jun Xiang, Chun Yu Lu, Xiao Xing Kong, Jian Mei Tian, Jiang Huai Wang, Jian Wang

**Affiliations:** 1 Pediatric Intensive Care Unit, Affiliated Children's Hospital, Soochow University, Suzhou, China; 2 Institute of Pediatric Research, Affiliated Children's Hospital, Soochow University, Suzhou, China; 3 Department of Pediatric Surgery, Affiliated Children's Hospital, Soochow University, Suzhou, China; 4 Department of the Infectious Disease, Affiliated Children’s Hospital, Soochow University, Suzhou, China; 5 Department of Academic Surgery, University College Cork, Cork University Hospital, Cork, Ireland

**Keywords:** Notch signaling, Subsets of T lymphocytes, Hand, Foot and mouth disease, Children

## Abstract

**Background:**

Hand, foot and mouth disease (HFMD), a virus-induced infectious disease that usually affects infants and children, has an increased incidence in China in recent years. This study attempted to investigate the role of the Notch signaling pathway in the pathogenesis of HFMD.

**Methods:**

Eighty-two children diagnosed with HFMD were enrolled into this study. The HFMD group was further divided into the uncomplicated HFMD and HFMD with encephalitis groups. The control group included 40 children who underwent elective surgery for treatment of inguinal hernias.

**Results:**

Children with HFMD displayed significantly reduced CD3^+^, CD3^+^CD4^+^ and CD3^+^CD8^+^ cell subsets, but substantially enhanced CD3^−^CD19^+^ cell subset (*p* < 0.05 versus control subjects). The expression levels of Notch ligands Dll1 and Dll4 in the peripheral blood of the HFMD group were significantly higher than those in the control group (*p* < 0.05). There were statistically significant differences in CD3^+^, CD3^+^CD4^+^ and CD3^−^CD19^+^ cell subsets, but not in Notch ligand expression, between the uncomplicated HFMD and HFMD with encephalitis groups. Dll4 expression in HFMD subjects correlated negatively with the CD3^+^ and CD3^+^CD8^+^ cell subsets (*p* < 0.05), but positively with the CD3^−^CD19^+^ cell subset (*p* < 0.05). Furthermore, Dll4 expression in HFMD with encephalitis subjects correlated positively with total white blood cell (WBC) counts and total protein contents in cerebrospinal fluid (CSF) (*p* < 0.05).

**Conclusions:**

The Notch ligand Dll4 exhibits a strong correlation with the CD3^+^, CD3^+^CD8^+^ and CD3^−^CD19^+^ cell subsets in children with HFMD, indicating that the Notch signaling may be involved in the development of HFMD by affecting the number and status of peripheral lymphocytes.

## Background

Hand, foot and mouth disease (HFMD) is a common infectious disease worldwide, which usually affects infants and children. In recent years, HFMD has become increasingly prevalent in the Asia-pacific region, particularly in China. HFMD is contagious and can be lethal in infants and children with severe cases. In China, there were 1,619,706 HFMD cases diagnosed with 509 deaths in 2011, 2,168,737 HFMD cases diagnosed with 567 deaths in 2012, and 1,828,377 HFMD cases diagnosed with 252 deaths in 2013, respectively [1,2]. The major pathogens of HFMD in these cases were enterovirus 71 (EV71) and coxsackie A virus A16 (CoxA 16) [1,2], but the precise mechanism (s) for the pathogenesis of HFMD has not yet been fully elucidated. Previous studies reported that children with HFMD displayed substantial immune disorders [3,4].

Notch signaling can promote or suppress cell proliferation, cell death, acquisition of specific cell fates, or activation of differentiation programs in a context-dependent manner, thus controlling cell fate and tissue homeostasis [5]. It has been shown that the Notch signaling pathway links both the innate immunity and the adaptive immunity, and affects the differentiation and development of T cells, B cells and natural killer (NK) cells, thereby playing an important role in the activation of host immune system against infectious disease [6-8]. Furthermore, Notch ligands Dll1 and Dll4 are both involved in the initiation of an anti-viral response [9,10]. While type-I IFN-induced Dll1 expression on macrophages plays a critical role in preventing influenza A virus infection [9], Dll4 appears to limit physiologic and pathologic changes in the lung during respiratory syncytial virus infection by modulating the Th2 response [10]. However, it is unclear whether the Notch signaling is involved in the pathogenesis of HFMD. To our knowledge, there has been no previous work reported on the role of the Notch signaling pathway in HFMD. In the present study, we attempted to identify correlations between the Notch signaling pathway and the immune status in pediatric patients with HFMD by assessing expression levels of Notch ligands and subsets of lymphocytes in the peripheral blood collected from children with HFMD. Our findings suggest a possible role for the Notch signaling pathway in HFMD.

## Methods

### Study population

A total of 82 pediatric patients who were admitted into the infection ward and Pediatric Intensive Care Unit of Affiliated Children’s Hospital, Soochow University, Suzhou, China and diagnosed with HFMD between June 2012 and December 2012 were recruited into this study. Among 82 cases, 42 cases with no complications were included in the uncomplicated HFMD group and 40 cases with encephalitis were included in the HFMD with encephalitis group. There were 26 males and 16 females in the uncomplicated group with an average age of 2.23 years ranging from 0.33 to 7 years and 24 males and 16 females in the encephalitis group with an average age of 2.6 years ranging from 0.75 to 9 years. The control group was comprised of 40 children (35 males and 5 females) with average age of 5.33 years ranging from 0.25 to 14 years who were scheduled for elective surgery of inguinal hernia repair. This study was approved by the Institutional Research Ethics Committee of Affiliated Children’s Hospital and Soochow University for clinical investigation, and the written informed consent was obtained from all study participants and/or their parents or guardians before enrollment. All experiments and procedures followed were conducted in accordance with the principles of the Declaration of Helsinki involving human subjects.

The diagnosis of HFMD and HFMD with encephalitis was based on the WHO diagnostic criteria [11]. Symptoms in HFMD children include fever and rashes (maculopapule, papules and small herpes) located on the hands, feet, mouth and buttocks, potentially accompanied by coughing, runny nose and lack of appetite. HFMD children complicated with encephalitis could display meningitis, encephalitis, poliomyelitis-like syndrome and encephalomyelitis symptoms and signs including weakness, lethargy, hyperarousal, headache, vomiting, dysphoria, shaky limbs, acute limb weakness and stiff neck, etc. Analysis of cerebrospinal fluid (CSF) in children with HFMD subjects revealed aseptic meningitis changes. Pediatric Risk of Mortality III (PRISM III) [12] was used to assess the disease severity in children with HFMD.

### Real-time qPCR

Real-time quantitative RT-PCR (q-PCR) was used to detect the expression levels of Notch ligands Dll1, Dll4, Jagged1 and Jagged2 in the peripheral blood. Total RNA was extracted using TRIzol (Invitrogen) and the single-stranded cDNA was synthesized using M-MLV reverse transcriptase (Invitrogen). Real-time qPCR was performed with the SYBR Green PCR Mix on a LightCycler System (Roche). The primers sequences used were hJAG1 sense-5’-AATGGTTATCGCTGTATCTG-3’ and antisense-5’-TCACTGGCACGGTTGTAG-3’, hJAG2 sense-5’-AGTTCCAGTGCGATGCCTACA-3’ and antisense-5’-GCTACAGCGATACCCGTTGAT-3’, hDLL1 sense-5’-GGGTCATCCTTGTCCTCAT-3’ and antisense-5’-CTTGGTGTCACGCTTGCT-3’, hDLL4 sense-5’-ACAGCCTATCTGTCTTTCGG-3’ and antisense-5’-GGCAGTGGTAGCCATCCT-3’ and glyceraldehyde 3-phosphate dehydrogenase (GAPDH), sense-5’-AAGCTCACTGGCATGGCCTT-3’ and antisense-5’-CTCTCTTCCTCTTGTGCTCTT G-3’. The transcript abundance was calculated using the ΔΔCt method, and the mRNA expression level of each Notch ligand was the ratio of normalized mean of GAPDH.

### FACScan analysis

Heparinized blood samples collected from different groups were dual- or triple-stained with anti-human CD3 (clone UCHT1, Beckman Coulter, Fullerton, CA), anti-human CD4 (clone SFCI12T4D11, Beckman Coulter), anti-human CD8 (clone SFCI21Thy2D3, Beckman Coulter), anti-human CD16 (clone 3G8, Beckman Coulter), anti-human CD19 (clone 89B, Beckman Coulter) and anti-human CD56 (clone N901, Beckman Coulter) mAbs conjugated with phycoerythrin (PE), fluorescein isothiocyanate (FITC) or phycoerythrin-Texas Red (ECD). PE-, FITC- or ECD-conjugated anti-human isotype-matched mAbs (Beckman Coulter) were used as negative controls. Erythrocytes were lysed with OptiLyse C (Beckman Coulter). FACScan analysis was performed for at least 10,000 events for detection of lymphocyte subsets in the peripheral blood including CD3^+^, CD3^+^CD4^+^, CD3^+^CD8^+^, CD3^−^CD19^+^ and CD3^−^CD16^+^CD56^+^ cells on a Coulter FC500 flow cytometer (Beckman Coulter) equipped with EXPO32 software (Beckman Coulter).

### Total WBC counting and protein measurement in CSF

CSF samples were collected from subjects in HFMD with encephalitis group. A 100 μl CSF sample was used to determine total WBC counts using a haemacytometer. Total protein contents in CSF samples were assessed by micro pyrogallol red colorimetric method.

### Statistical analysis

Results are expressed as mean ± standard deviation (SD). Data were collected and analyzed using GraphPad software, version 5.01 (Prism, La Jolla, CA). All data were tested for normal distribution and homogeneity of variance. The unpaired student’s t-test was used to compare the means between two groups. The unpaired student’s t-test with Welch’s correction was used to analyze non-normally distributed values. The Spearman rank correlation coefficient test was used to assess correlations between Notch ligand expression and lymphocyte subsets in the peripheral blood. A *p*-value of less than 0.05 was considered to be statistically significant.

## Results

### Alterations in peripheral lymphocyte subsets in HFMD subjects

The numbers of CD3^+^ (*p* = 0.014), CD3^+^CD4^+^ (*p* = 0.006) and CD3^+^CD8^+^ (*p* = 0.001) cells in the peripheral blood of the HFMD group were significantly lower than those in the control group, whereas the number of CD3^−^CD19^+^ cells (*p* = 0.007) in the peripheral blood of the HFMD group was significantly higher than that in the control group (Table 1). No significant difference in the number of peripheral CD3^−^CD16^+^CD56^+^ cells was found between the HFMD and control groups (Table 1). The numbers of peripheral CD3^+^ (*p* = 0.023) and CD3^+^CD4^+^ (*p* = 0.039) cells in the uncomplicated HFMD group were substantially higher than those in the HFMD with encephalitis group, whereas the number of peripheral CD3^−^CD19^+^ cells (*p* = 0.001) was significantly lower in the uncomplicated HFMD group compared to the HFMD with encephalitis group (Table 2). There were no significant differences in the numbers of peripheral CD3^+^CD8^+^ and CD3^−^CD16^+^CD56^+^ cells found between the uncomplicated HFMD and HFMD with encephalitis groups (Table 2).

**Table 1:** Comparison of lymphocyte subsets in the peripheral blood between the control group (n = 40) and the HFMD group (n = 82)

**Lymphocyte subsets (%)**	**Control**	**HFMD**	***p *** **values**
CD3^+^	59.94 ± 6.41	56.52 ± 9.83	0.014
CD3^+^CD4^+^	32.15 ± 6.31	28.57 ± 8.36	0.006
CD3^+^CD8+	26.42 ± 5.65	22.70 ± 6.59	0.001
CD3^−^CD19^+^	21.41 ± 6.38	24.93 ± 8.50	0.007
CD3^−^CD16^+^CD56^+^	17.08 ± 5.99	15.82 ± 8.10	0.306

Data are mean ± SD. Statistical significance was evaluated by unpaired student’s t-test.

**Table 2:** Comparison of lymphocyte subsets in the peripheral blood between the uncomplicated HFMD group (n = 42) and the HFMD with encephalitis group (n = 40)

**Lymphocyte subsets (%)**	**Uncomplicated**	**Encephalitis**	***p *** **values**
CD3^+^	58.42 ± 8.99	54.21 ± 10.40	0.023
CD3^+^CD4^+^	30.04 ± 8.87	26.79 ± 7.40	0.039
CD3^+^CD8+	23.14 ± 6.42	22.00 ± 6.88	0.443
CD3^−^CD19^+^	22.62 ± 7.87	27.74 ± 8.46	0.001
CD3^−^CD16^+^CD56^+^	16.40 ± 7.38	15.13 ± 8.92	0.412

Data are mean ± SD. Statistical significance was evaluated by unpaired student’s t-test.

### Levels of Notch ligand expression between HFMD and control subjects

**Figure 1:**
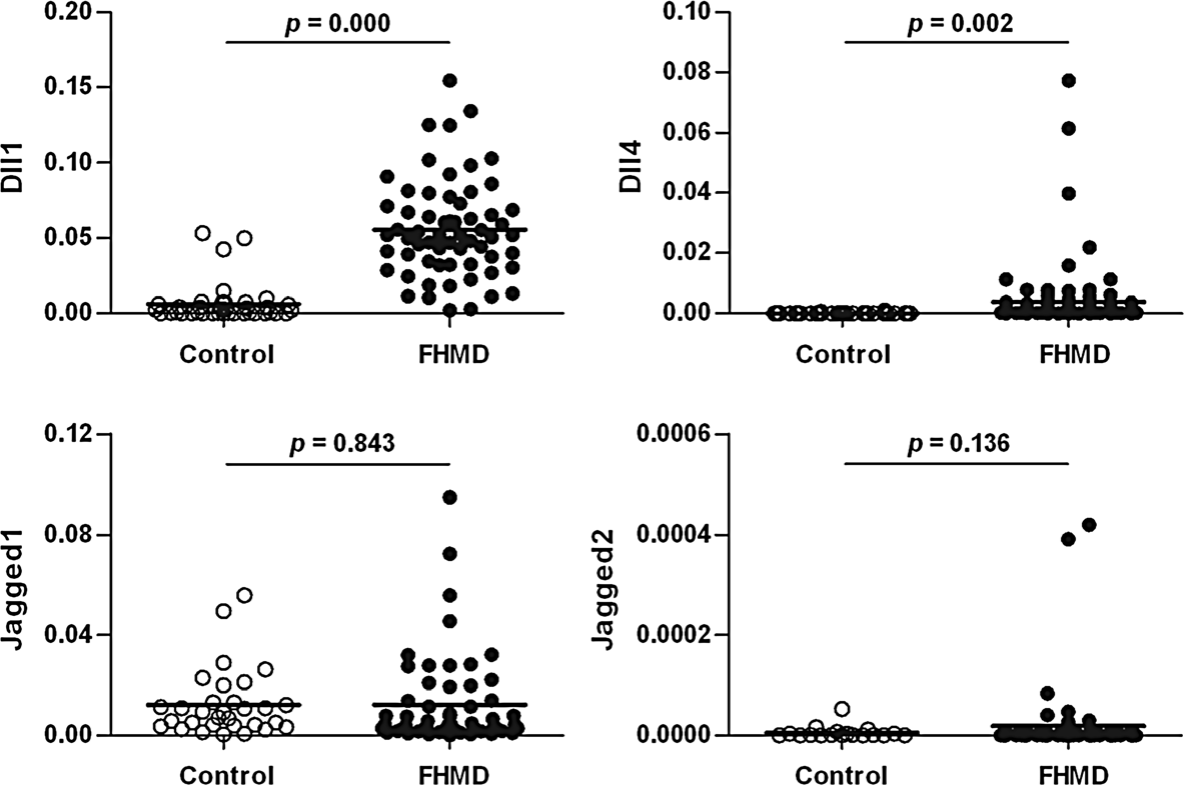
**Comparison of the expression levels of Notch ligands Dll1, Dll4, Jagged1 and Jagged2 in the peripheral blood between the control group (n = 40) and the HFMD group (n = 82).** The mRNA expression levels of Dll1, Dll4, Jagged1 and Jagged2 were assessed by real-time q-PCR and normalized with GAPDH as described in the Methods. Each dot represents individual case and the horizontal line represents the mean. Statistical significance was evaluated by unpaired student’s t-test with Welch’s correction.

**Figure 2:**
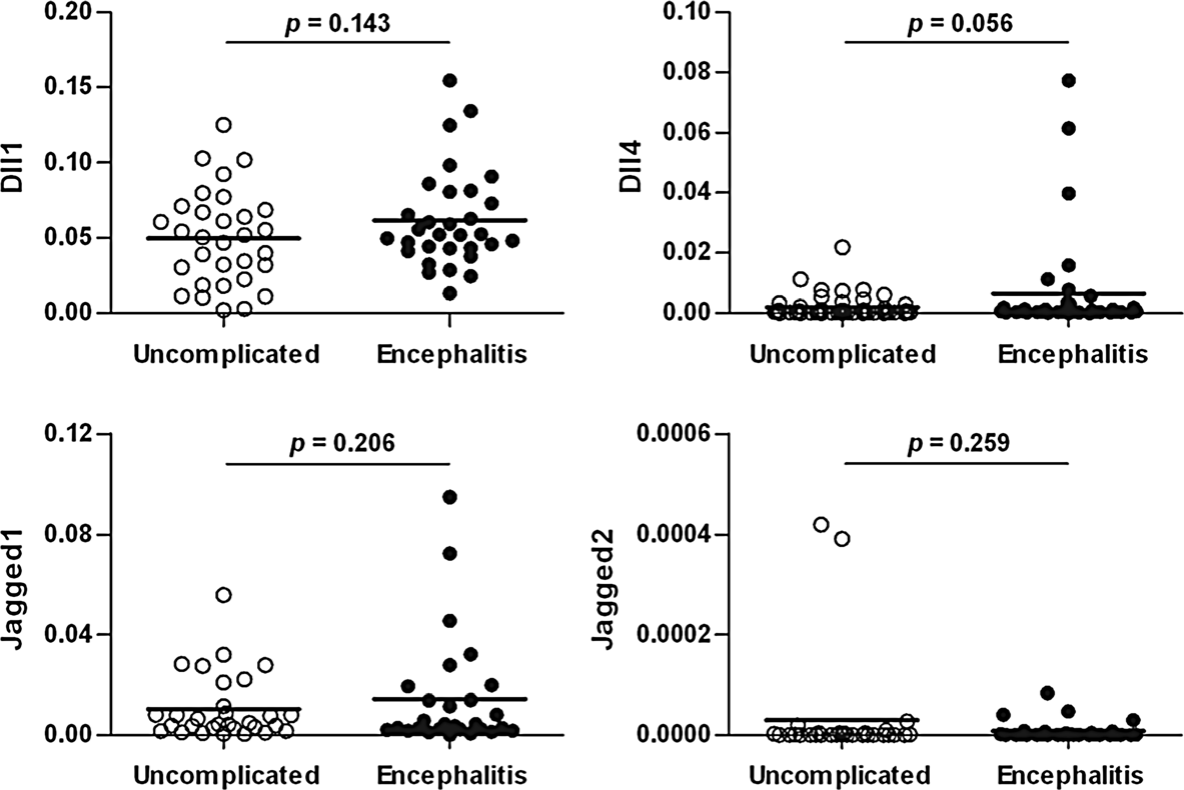
**Comparison of the expression levels of Notch ligands Dll1, Dll4, Jagged1 and Jagged2 in the peripheral blood between the uncomplicated HFMD group (n = 42) and the HFMD with encephalitis group (n = 40).** The mRNA expression levels of Dll1, Dll4, Jagged1 and Jagged2 were assessed by real-time q-PCR and normalized with GAPDH as described in the Methods. Each dot represents individual case and the horizontal line represents the mean. Statistical significance was evaluated by unpaired student’s t-test with Welch’s correction.

The expression levels of Notch ligands Dll1 (*p* = 0.000) and Dll4 (*p* = 0.002) in the peripheral blood of the HFMD group were significantly higher than those of the control group (Figure 1); however, there were no significant differences in Jagged1 and Jagged2 expression levels found between the HFMD and control groups (Figure 1). There were also no significant differences in Dll1, Dll4, Jagged1 and Jagged2 expression levels found between the uncomplicated HFMD and HFMD with encephalitis groups (Figure 2).

### Correlations between Dll4 expression and peripheral lymphocyte subsets

**Figure 3:**
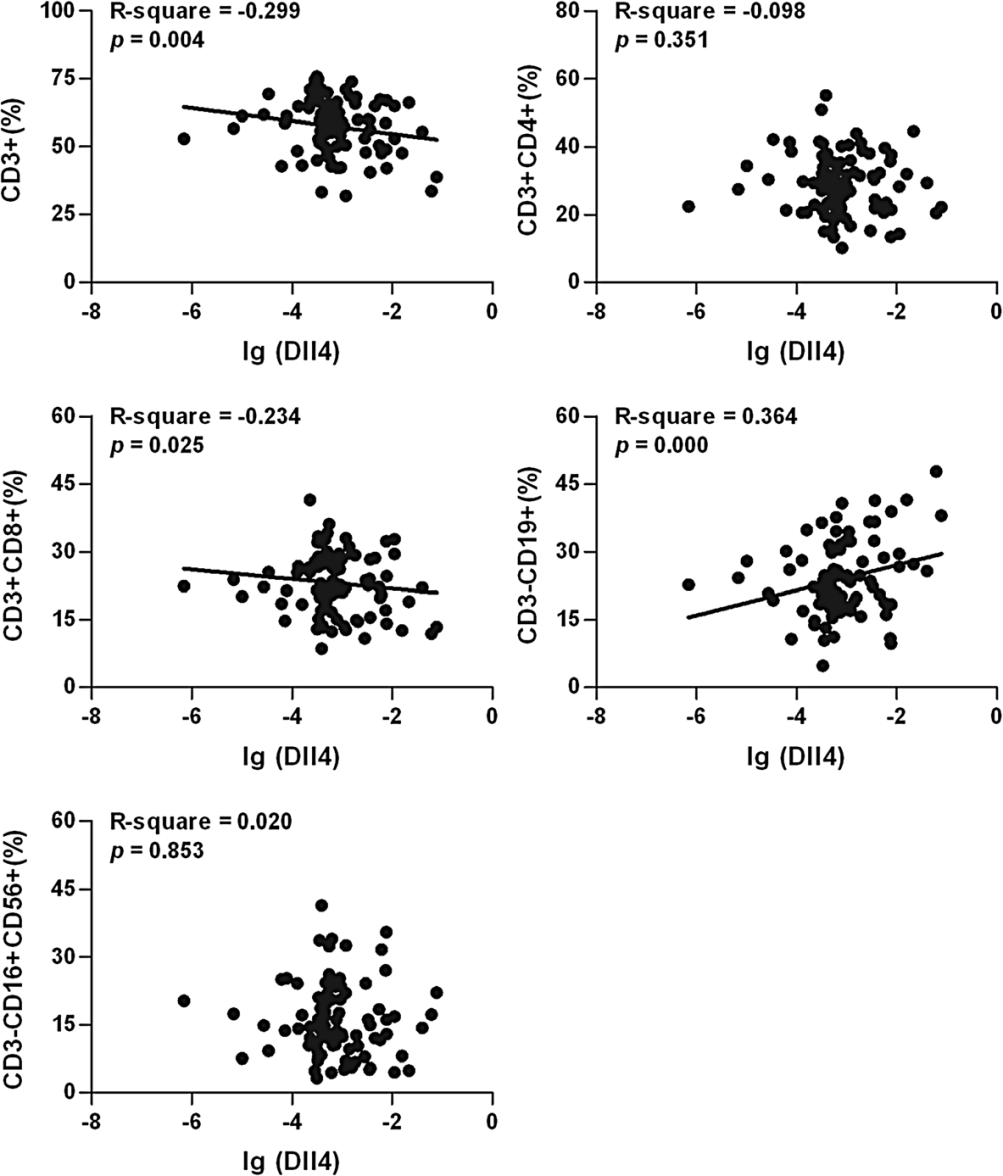
**Correlation between the Dll4 expression levels and the CD3**^**+**^**, CD3**^**+**^**CD4**^**+**^**, CD3**^**+**^**CD8**^**+**^**, CD3**^**−**^**CD19**^**+**^**, or CD3**^**−**^**CD16 + CD56**^**+ **^**cell subsets in HFMD patients (n = 82).** Dll4 mRNA expression and lymphocyte subsets were assessed by real-time q-PCR and FACScan analysis respectively, as described in the Methods. Each dot represents individual case and each line indicates the best-fit line. R^2^ values were calculated by Spearman rank correlation coefficient test.

The expression levels of Notch ligand Dll4 in the peripheral blood of the HFMD group correlated negatively with the numbers of peripheral CD3^+^ (R-square = −0.299, *p* = 0.004) and CD3^+^CD8^+^ (R-square = −0.234, *p* = 0.025) lymphocytes, but correlated positively with the number of peripheral CD3^−^CD19^+^ lymphocyte (R-square = 0.364, *p* = 0.000) (Figure 3). There were no correlations found between Dll4 levels and peripheral CD3^+^CD4^+^ (R-square = −0.098, *p* = 0.351) and CD3^−^CD16^+^CD56^+^ (R-square = 0.020, *p* = 0.853) cell counts (Figure 3). By contrast, the expression levels of Dll1 did not correlated with the numbers of peripheral lymphocyte subsets (data not shown).

### Association between Dll4 expression levels and HFMD

**Figure 4:**
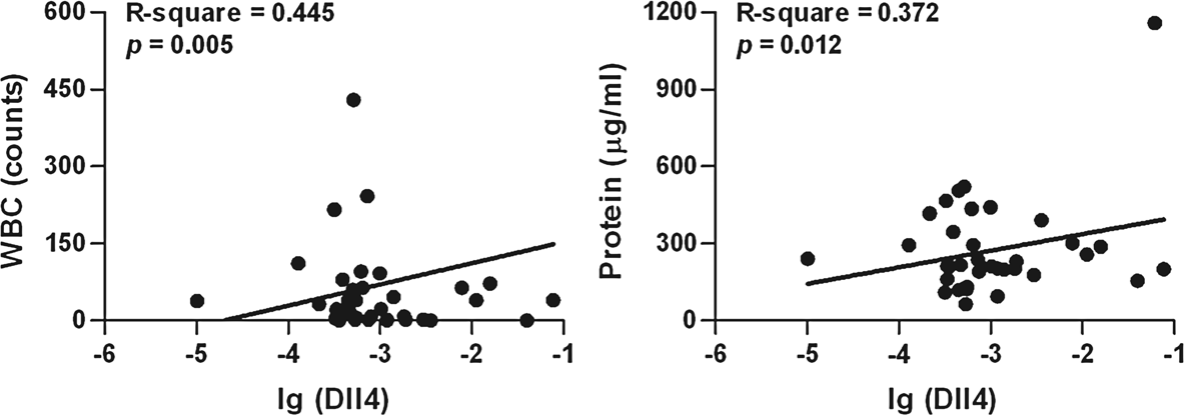
**Association between the Dll4 expression levels and CSF WBC counts or CSF protein contents in HFMD patients with encephalitis (n = 40).** Dll4 mRNA expression was assessed by real-time q-PCR. CSF WBC counts and protein contents were determined as described in the Methods. Each dot represents individual case and each line indicates the best-fit line. R^2^ values were calculated by Spearman rank correlation coefficient test.

A positive correlation was found in the HFMD with encephalitis group between Dll4 expression levels in the peripheral blood and total WBC counts in CSF (R-square = 0.445, *p* = 0.005) as well as between Dll4 expression levels in the peripheral blood and total protein contents in CSF (R-square = 0.372, *p* = 0.012) (Figure 4). However, the expression levels of Dll4 in the peripheral blood of HFMD subjects did not correlate significantly with the duration of fever, length of hospital stay, the biochemical markers CRP, Glu, Alt, Ast, CK and CK-MB, and the PRISM III score (data not shown).

## Discussion

HFMD is a virus-induced infectious disease, which can lead to serious consequences especially in infants and children. Several studies have shown that children with HFMD undergo significant alterations in their immune status [3,4]. However, the precise mechanism (s) responsible for altered immune functions in patients with HFMD has not yet been fully clarified.

In the present study, we found that children with HFMD displayed significant decreases in their peripheral CD3^+^, CD3^+^CD4^+^ and CD3^+^CD8^+^ cell subsets, but had a substantial increase in their peripheral CD3^−^CD19^+^ cell subset. Moreover, children in the HFMD with encephalitis group showed further reduction in the CD3^+^ and CD3^+^CD4^+^ cell subsets and elevation in the CD3^−^CD19^+^ cell subset compared to children in the uncomplicated HFMD group. These results are consistent with previously reported findings in the mainland of China and Taiwan [3,4]. We also found significant increases in the expression levels of Notch ligands Dll1 and Dll4 in the peripheral blood of children with HFMD, suggesting that Notch signaling might be initiated and activated during HFMD via the engagement of upregulated Notch ligand Dll1 or Dll4 with Notch receptors, which subsequently affects the differentiation and development of T and B lymphocytes.

It is unclear whether the Notch signaling is associated with changes in the immune status observed in children with HFMD. Previous studies have shown that the Notch signaling pathway possesses a crucial role in differentiations of a variety of immune cells [13,14]. Mukuherjee et al. [15] found that the Notch ligand Dll4 promoted T-cell differentiation through increased expression of IL-17 and RORγ T. Schaller et al. [10] also found that Dll4 expression on bone marrow-derived dendritic cells (DCs) increased significantly after infection of mice with respiratory syncytial virus, accompanied by increased secretion of Th2 cytokines and reduced production of INF-γ. In the present study, we performed a correlation analysis of the cell counts of different lymphocyte subsets with Notch ligand expression levels in the peripheral blood of children with HFMD. Our data demonstrated that peripheral lymphocyte subsets had statistically significant correlations with the Notch ligand Dll4 expression. The Dll4 expression levels showed negative correlations with CD3^+^ and CD3^+^CD8^+^ cell subsets, but a positive correlation with CD3^−^CD19^+^ cell subset that has the B lymphocyte surface antigen characteristics, suggesting that the up-regulated Dll4 expression may be associated with a relatively inhibited status of CD3^+^ lymphocytes and a relatively activated status of CD3^−^CD19^+^ lymphocytes observed in children with HFMD. It has been reported that the prognosis of sepsis correlates closely with the numbers of NK cells, CD3^+^, CD3^+^CD4^+^ and CD3^+^CD8^+^ lymphocytes [16]. Therefore, it is possible that the Notch signaling pathway also affects HFMD prognosis by interfering with the number and status of these immune cells.

In the present study, we further showed that the Dll4 expression levels in the peripheral blood correlated positively with total WBC counts and total protein contents in CSF, but there were no correlations of the Dll4 expression levels with the duration of fever, length of hospital stay, the biochemical markers CRP, Glu, Alt, Ast, CK and CK-MB, and the PRISM III score. The total WBC counts and total protein contents in CSF are critical for the diagnosis of encephalitis and are also indicators for the degree of brain inflammation [17,18]. However, we did not observe any significant differences in Notch ligand expression such as the Dll4 level between the uncomplicated HFMD group and the HFMD with encephalitis group. Further work is needed to verify whether Dll4 can be used as a clinical indicator for determination of the severity of HFMD.

A crucial cause of death in children with severe HFMD is the neurogenic pulmonary edema. Infection with EV71 generates viremia or penetrates directly into the central nervous system where it mainly affects the brainstem, which leads to disorders of the autonomic nerve function and stimulation of sympathetic nerve system [19-22]. The over-activated sympathetic nerve system causes a massive release of catecholamines [23], resulting in systemic vasoconstriction and a shift of blood flow from the systemic circulation to the pulmonary circulation, which finally leads to the development of neurogenic pulmonary edema. It has been reported that activation of angiopoietin 2 and Dll4 can promote regeneration of the damaged dopamine neurons, thereby resulting in catecholamine such as dopamine release [24]. Children with severe HFMD may also release significant amounts of catecholamine. In the present study, we further observed substantially up-regulated Notch ligand Dll4 (or activation of Dll4) in children with HFMD. Thus, our further work will examine whether pharmacological inhibition of Dll4 could attenuate catecholamine release, thereby reducing the incidence of neurogenic pulmonary edema in children with severe HFMD.

## Conclusions

Children with HFMD undergo significant changes in their immune status, and the Notch signaling pathway may play an important role in these changes. Furthermore, the Notch ligand Dll4 correlates strongly with the peripheral CD3^+^, CD3^+^CD8^+^ and CD3^−^CD19^+^ lymphocyte subsets, indicating that Dll4 may participate in the pathogenesis of HFMD by interfering with the number and status of these immune cells.

## Abbreviations

CoxA 16: Coxsackie A virus A16; CSF: Cerebrospinal fluid; DCs: Dendritic cells; ECD: phycoerythrin-texas red; EV71: Enterovirus 71; FITC: Fluorescein isothiocyanate; HFMD: Hand, foot and mouth disease; NK cells: Natural killer cells; PE: Phycoerythrin; PRISM III: Pediatric risk of mortality III; q-PCR: quantitative RT-PCR; WBC: White blood cells.

## Competing interests

The authors declare that they have no competing interests.

## Authors’ contributions

ZJB, YPL, JHW, JW conceived and designed the experiments. ZJB, YPL, JH, YJX, CYL performed the experiments. ZJB, YPL, XXK, JMT, JHW, JW analyzed the data. ZJB, YPL, JHW, JW wrote the paper. All authors read and approved the final manuscript.

## Pre-publication history

The pre-publication history for this paper can be accessed here:

http://www.biomedcentral.com/1471-2334/14/337/prepub
